# Novel Role of AaMYBC1 in Regulating *Actinidia arguta* Vine Architecture by Elongating Internode Based on Multi-Omics Analysis of Transgenic Tobacco

**DOI:** 10.3390/genes13050817

**Published:** 2022-05-03

**Authors:** Yukuo Li, Hailei Huang, Muhammad Abid, Hong Gu, Zhongping Cheng, Jinbao Fang, Xiujuan Qi

**Affiliations:** 1 Zhengzhou Fruit Research Institute, Chinese Academy of Agricultural Sciences, Zhengzhou 450009, China; liyukuo@caas.cn (Y.L.); 82101195082@caas.cn (H.H.); guhong@caas.cn (H.G.); fangjinbao@caas.cn (J.F.); 2 Lushan Botanical Garden, Chinese Academy of Sciences, Jiujiang 332900, China; muhammadabid@lsbg.cn; 3 Wuhan Botanical Garden, Chinese Academy of Sciences, Wuhan 430074, China; chenzp@wbgcas.cn

**Keywords:** AaMYBC1, transgenic tobacco, omics analysis, internode length, vine architecture, *Actinidia arguta*

## Abstract

The internode length affects the status of fruiting branches and shapes the vine architecture. MYB TFs (transcription factors) have been widely studied and reported to control many biological processes including secondary metabolism, abiotic stresses, growth and development, etc. However, the roles of MYB TFs in regulating internode length remain poorly understood. Here, we demonstrated that a secondary metabolism-related R2R3-MYB TF AaMYBC1 from Actinidia arguta was involved in the regulation of internode length by combined analysis of transcriptome and metabolome of transgenic tobacco plants. The metabolome analysis of OE (over-expressed tobacco) and WT (wild-typed tobacco) showed that there were a total of 1000 metabolites, 176 of which had significant differences. A key metabolite pme1651 annotated as indole 3-acetic acid belonged to phytohormone that was involved in internode length regulation. The RNA-seq analysis presented 446 differentially expressed genes (DEGs) between OE and WT, 14 of which were common DEGs in KEGG and GO enrichment. Through the combined analysis of metabolome and transcriptome in transgenic and wild-type tobacco, three key genes including two SAUR and a GH3 gene were possibly involved in internode elongation. Finally, a regulatory module was deduced to show the role of AaMYBC1 in internode elongation. Our results proposed a molecular mechanism of AaMYBC1 regulating internode length by mediated auxin signaling, implying the potential role in regulating the vine architecture.

## 1. Introduction

During past decades, fruit quality and yield have been considered two important traits for fruit tree breeding. Studies on fruit quality, including fruit size [1,2], coloring [3,4,5], sweetness [6,7], acidity [8,9], aroma [10,11] and functional substances [12], as well as fruit growth, development, maturity and softening, have been widely reported [13,14,15,16]. The regulatory mechanisms underlying fruit yield in plants are less studied due to multiple influencing factors including variety selection, irrigation, fertilization, cultural practices, the complexity of regulatory mechanisms, and the difficulty in establishing a suitable research platform. While the cultural and management practices of orchards and a reasonable tree architecture are key players in determining fruit quality.

Kiwifruit belongs to the Actinidiaceae, genus *Actinidia*, which possesses a rich germplasm resource, including 54 species and 21 varieties [17]. Recently, the *Actinidia arguta* (Siebold and Zuccarini) Planchon ex Miquel (*A. arguta*), a kind of mini berry, has developed into the second-largest cultivated species after *A. chinensis* [18]. The *A. arguta* has gained popularity in the consumer market due to hairless skin, edible pericarp, no process of post ripening, and ready-to-eat fruit [19]. Unfortunately, the ordinary quality of fruit and the low fruit yield (many *A. arguta* orchards produce less than 22,500 kg per hectare), has halted the large-scale commercial development of *A. arguta* orchards. Therefore, some reasonable cultural and management practices are required to improve the *A. arguta* vine architecture. The vine architecture affects plant growth and fruit quality through efficient of light energy utilization and carbon assimilation, reasonable distribution of photosynthetic products, high fruit yield to produce high economic benefits, and convenient management of the orchard [20]. Internode length plays an essential role in shaping the vine architecture. Therefore, it is prudent to dissect molecular regulatory mechanisms underlying internode length to achieve the goal of shaping better vine architecture.

Previously, the studies on internode length were mainly focused on the plant height in crops, i.e., soybean [21,22], maize [23], wheat [24], rice [25], etc. The endogenous production of plant growth hormones (PGR) in small proportion is indispensable in the regulation of internode length. The GA (gibberellic acid) is an important PGR that regulated plant height by changing GA synthesis, metabolism or signaling transduction [26]. The key DELLA protein-encoding genes, *Rht-1*, *Rht-Bit* and *Rht-D1b* that compose allele variants, confer the DELLA as a negative responsiveness in GA signaling, which in turn reduced plant growth [27,28]. The loss of function in GA20_OX_2, a key GA biosynthesis enzyme, because of mutations in gene *sd1* and *sdw1* decreased the GA levels in rice and barley [29,30]. Additionally, a GA2-oxidase encoding gene *Rht18* reduced GA biosynthesis by converting active GA into inactive forms [31]. Besides GA, other plant hormones are also involved in internode regulation. Auxin is known as a shoot-to-root hormone that regulates several agricultural traits including internode length, shoot branching plant height, etc. [32]. PIN (PIN-FORMED)/AUX1/ABCB (p-glycoprotein ABC transports) family members-mediated auxin polar transport controlled the plant height by establishing an auxin gradient [33,34,35]. A serine/threonine-protein kinase encoding gene *PID* belongs to the AGCVIIIa kinase family, which catalyzes auxin efflux by PIN localization and/or phosphorylation [36,37]. The OsPIN2/OsPIN5 and ZmPIZ1a reduced internode length and plant height by accelerating auxin transport from shoots to roots in rice and maize, respectively [38,39]. These results suggested the key role of plant hormones including GA and auxin in the regulation of internode length. However, the molecular mechanisms underlying the formation of tree architecture caused by internode length are rarely studied in fruit trees. In the past, researchers preliminarily explored the relation between tree architecture and internode length by combining genome-wide analysis with a functional and structural plant model in apples without identifying the genes controlling internode length [40]. The application of some pruning strategies in kiwifruit (*Actinidia deliciosa* cv. Hayward) has been reported to affect vine architecture, fruit quality, and fruit yield [41]. The *Actinidia arguta* was found to be more productive on 1-year-old canes regardless of cane origin or length [42], which provided useful insights into orchard production. However, there is no reported work available on molecular mechanism and regulatory genes involved in internodal length to form kiwifruit vine architecture.

In a previous study, we found that a fruit quality-related R2R3-MYB transcription factor AaMYBC1 might play an important role in the regulation of internode length. Therefore, we performed a heterologous transformation of AaMYBC1 in tobacco plants to observe its regulatory role in the internode length of kiwifruit vine. Through multi-omics analysis of different tobacco internodes, we found that AaMYBC1 regulated internode length by mediating auxin signals. Our findings will help plant breeders to improve fruit quality and yield by controlling kiwifruit vine architecture through biotechnology techniques.

## 2. Materials and Methods

### 2.1. AaMYBC1 Cloning and Vector Construction

AaMYBC1 sequence was amplified from *A. arguta* by specific pair of primers (Forward primer: 5′-**GGAGAGGACACGCTCGAG**ATGGGGAGAAGCCGATGT-3′; Reverse primer: 5′-**TTAAAGCAGGACTCTAGA**CTACCCGAAACCTTGGTGATT-3′, the base pairs in boldface are homologous sequence from overexpression vector). The target gene was detected by 1% agarose gel electrophoresis and the product was recovered using Gel Extraction Kit (Bioteke corporation, Beijing, China). The homologous recombination method with double restriction enzyme digestion (*Xho* I and *Xba* I) was adopted to construct a recombinant vector by introducing the AaMYBC1 gene sequence into the pART-CAM overexpression vector regulated by the 35S promoter. The specific procedures for preparing the reaction mixture was as follows: the reaction system containing Tango buffer 4.0 µL, *Xho* I 1 µL, *Xba* I 1 *Xba* I, pART-CAM plasmid 1 µg, ddH_2_O up to a total volume of 20 µL was run at 37 °C for 2.5 h, and then the recombinant DNA fragment was recovered by using Gel Extraction Kit (Bioteke corporation, Beijing, China). The *AaMYBC1* gene fragment and linearized pART-CAM vector were added to the EP tube for running recombination reaction at 37 °C for 30 min to form the pART-CAM::*AaMYBC1* recombinant plasmid. The recombinant vector was transformed into DH5α component cells and the successful transformation of the vector was identified by running bacterial colonies on PCR and the sequence accuracy was confirmed by Sanger sequencing. The AaMYBC1 gene information was deposited in the NCBI database under GenBank accession number MN249175.

### 2.2. Transformation of Tobacco Plants with AaMYBC1

The overexpression vector 35S:pART-CAM::AaMYBC1 was transformed into *Agrobacterium tumefaciens* strain GV3101. The specific transformation protocol was as follows: 10 µL of recombinant plasmid pART-CAM::AaMYBC1 was added into the semi-melted *Agrobacterium tumefaciens* GV3101, gently mixed and placed on ice for 10 min, followed by quick freezing in liquid nitrogen for 5 min, water bath at 37 °C for 5 min, and ice bath for 5 min. Finally, the bacterial culture was added to 800 µL LB liquid medium without any antibiotic and shaken at 28 °C for 4 h. The culture was centrifuged at 5000 rpm for 1 min and the resulting bacterial cells were cultured on solid medium plates placed upside down under 28 °C after re-suspension. The single colony was selected for PCR detection and Sanger sequencing. The small square tobacco SR1 leaves without margin and the main vein from sterile 1–2 months old seedlings were infiltrated with a bacterial culture containing 35S:AaMYBC1, and then co-cultured for 2–3 days on MS medium containing 6-BA 1 mg/L with pH 5.8 under dark conditions. Then, the explants were transferred to a regeneration MS medium containing 6-BA 1mg/L, Timentin 300 mg/L, and Kan 100 mg/L with pH 5.8. The resistant shoots with 2 cm height were cut off and transferred to a 1/2 MS rooting medium containing Timentin 300 mg/L, IAA 0.5 mg/L and Kan 100 mg/L with pH 5.8. The fully grown transgenic tobacco plants were transferred into pots and places in the greenhouse under suitable growth conditions.

### 2.3. Metabolome Analysis for Transgenic Tobacco Plants

The samples from transgenic and wild-type tobacco plant internodes freeze-dried by vacuum freeze-dryer were crushed with a mixer mill for 15 min at 30 Hz. The 100 mg powder from samples was dissolved in 70% methanol solution, and then rotated for 30 s every 30 min for 6 times in total. After overnight refrigeration at 4 °C, the samples were centrifuged for 10 min at 12,000 rpm to obtain extracts that were used for subsequent metabolome analysis with UPLC-ESI-MS/MS system (UPLC, SHIMADZU Nexera X2, Kyoto, Japan; MS, Applied Biosystems 4500 Q TRAP, Waltham, MA, USA).

The metabolome data were subjected to PCA (principal component analysis) by windows-based R software. Through PCA of samples, we can get the overall information about metabolic difference between OE and WT groups, and the variability between samples in the OE group or WT group. PCA results showed the trend of metabolome separation between OE and WT groups and suggested whether there were differences in metabolome between the OE or WT samples [43,44]. In addition, the HCA (hierarchical cluster analysis) for samples and metabolites and PCC (Pearson correlation coefficients) for different samples were presented and calculated by heatmap for R (v1.0.12) and the Hmisc for R (v3.5.1/v4.4.0), respectively (University of Auckland, Auckland, New Zealand).

The OPLA-DA (Orthogonal Partial Least Squares-Discriminant Analysis) was carried out to identify significantly different metabolites between OE and WT groups based on fold change >2 or <0.5 and VIP (Variable Importance in Projection) > 1 by using MetaboAnalystR package for R (v1.0.1) (University of Auckland, Auckland, New Zealand) [45]. The overfitting of metabolites in OPLA-DA was avoided by the permutation test. The annotation information of identified metabolites in enriched pathways was mapped by using an online KEGG database (Kyoto University, Kyoto, Japan) [46].

### 2.4. RNA-Seq Preparation, Data Obtaining and Processing

In order to ensure that the quality of RNA met the subsequent tests, after RNA extraction, the RNA degradation and purity were determined on 1% agarose gels and checked by NanoPhotometer^®^ spectrophotometer (IMPLEN, Palo Alto, CA, USA), respectively. The RNA concentration and integrity were measured using Qubit^®^ RNA Assay Kit in Qubit^®^2.0 Fluorometer (Life Technologies, Palo Alto, CA, USA) and assessed by the RNA Nano 6000 Assay Kit of the Bioanalyzer 2100 system (Agilent Technologies, Palo Alto, CA, USA).

A total of 1 µg RNA per sample was used for library preparations. The mediation, NEBNext^®^ UltraTM RNA Library Prep Kit for Illumina^®^ (NEB, San Diego, CA, USA), was used for the generation of sequencing library generation per sample. The specific recommendations were as follows: mRNA after purification from total RNA was used for the first-strand cDNA synthesis through random hexamer primer and M-MuLV Reverse Transcriptase, and then the second-strand cDNA was synthesized by using DNA Polymerase I and RNase H. The fragments in the library were purified with the AMPure XP system to choose cDNA fragments of 250–300 bp in length (Beckman Coulter, Beverly, MA, USA). Then, the PCR products were purified and the library was assessed by the AMPure XP system and Agilent Bioanalyzer 2100 system, respectively. The cDNA libraries were sequenced on the Illumina sequencing platform by Metware Biotechnology Co., Ltd. (Wuhan, China).

The original data was filtered with fastp (v 0.19.3) (HaploX, Shenzhen, Guangdong, China) to remove adapters and some low-quality reads (Q < 20) were also removed [47]. The clean reads were compared to the reference genome by using HISAT v2.1.0 (JHU, Baltimore, MD, USA) to obtain annotation files [48]. The new transcripts were predicted by StringTie v1.3.4d (JHU, Baltimore, MD, USA) [49], which could splice transcripts more accurately and completely at a faster speed. After performing the gene alignment, the quantification of gene expression levels in FPKM (Fragments Per Kilobase of transcript per Million fragments mapped) was calculated by featureCounts v1.6.2 (The University of Melbourne, Parkville, Australia) [50,51]. Differential expression of genes between two groups was analyzed by the DESeq2 v1.22.1 (EMBL, Heidelberg, Germany) [52,53], and the *p*-value was corrected by using Benjamini and Hochberg method. The threshold for differential expression of genes was indicated by corrected *p*-value and log2foldchange. Differential gene enrichment analysis was performed under the premise of the hypergeometric test, in which distribution test was conducted by unit of pathway for KEGG and based on the GO term for GO, respectively [54].

### 2.5. Combined Analysis of Transcriptome and Metabolome

The transcriptome is a technology to reveal the expression rule of mRNA in specific samples at a specific stage, while metabolome is to study the accumulation changing of metabolites in specific samples at a specific stages. To a certain extent both technological ways can provide some useful information for explaining biological traits, but biological processes are complex and dynamically changeable, and it is difficult to systematically explain the trait occurrence through a single omics. Therefore, it is necessary to conduct combined analysis of transcriptome and metabolome to reveal the biological mechanism. Firstly, we mapped the significantly different metabolites and genes from the same groups to the KEGG pathways to better understand the relationship between metabolites and genes involved in the same biological processes. Then, a histogram was drawn to show the degree of pathway enrichment for differential metabolites and genes. The Pearson correlation coefficients was carried out between genes and metabolites to find the association between differentially expressed genes and significantly different metabolites by setting the Pearson correlation coefficient value >0.8. Additionally, the correlation analysis results were used to perform correlation coefficient cluster analysis and correlation network analysis. Finally, the canonical correlation analysis was conducted to reveal the whole correlation between genes and metabolites from each pathways using the correlation relationship between comprehensive variable pairs. All these methods were applied to reveal the correlation between molecular level and metabolic level [55,56].

### 2.6. Statistical Analysis

All statistical analyses were carried out by IBM SPSS 20 (IBM, Armonk, NY, USA) and Graphs were visualized by GraphPad Prism (GraphPad Software Inc., San Diego, CA, USA). The mean differences for data were calculated by using Student’s *t*-test and the significance level was determined by *p* < 0.05. We used three biological replicates for all samples.

## 3. Results and Discussion

### 3.1. Phenotype, Length and Cytological Observation of Transgenic Tobacco Internode

**Figure 1:**
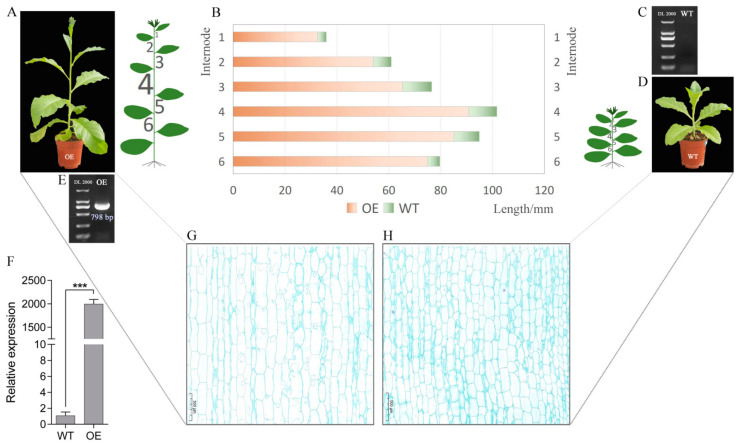
Analysis for internode length of transgenic and wild-type tobacco plants. (**A**) The phenotypes of transgenic tobacco SR1 overexpressing *AaMYBC1*. (**B**) Length of six internodes. The yellow and green bar charts represent OE and WT tobacco plants, respectively. The length was measured in millimeters. (**C**) PCR detection of WT tobacco. (**D**) The phenotype of wide typed tobacco SR1. (**E**) PCR detection of OE tobacco plants. (**F**) The expression level of *AaMYBC1* in OE and WT tobacco plants. The tobacco actin gene was used as internal control during the RT-qPCR experiment. Values are represented as ±SD of three biological replicates. The mean difference was declared significant at *p* < 0.001 and presented as “***”. (**G**) Cytological observation of internode from OE tobacco plants. (**H**) Cytological observation of internode from WT tobacco plants. Scale bars: 500 µm.

A total of 15 independent transgenic tobacco lines were obtained by stable genetic transformation. The transgenic tobaccos plants overexpressing AaMYBC1 exhibited excellent growth and development, and their internode length was significantly longer than that of the wild typed tobaccos plants (Figure 1A,D). Three positive lines were randomly selected to perform further analysis. We tested the expression level of *AaMYBC1* in OE and WT tobacco plants by RT-PCR and RT-qPCR analysis. The RT-PCR results showed that the transgenic lines had a 798 bp long specific bright band for *AaMYBC1* (Figure 1E), while no band was found for WT tobacco (Figure 1C). The RT-qPCR results showed that the expression level of *AaMYBC1* in OE tobacco lines thousand times higher than that of WT tobacco plants (Figure 1F), which indicated that the long internode phenotype in transgenic lines was particularly caused by higher expression of *AaMYBC1*. The length measurement for a total of six internodes from the middle of the plant showed that each internode was longer than that of the wild-type tobacco plant (Figure 1B). To explore the cytological causes of internode length, we observed the internode section and found that the cells of internode from transgenic plants were significantly elongated compared to the wild-type tobacco plants (Figure 1F,G).

### 3.2. Metabolome Analysis and Metabolite Identification

**Figure 2:**
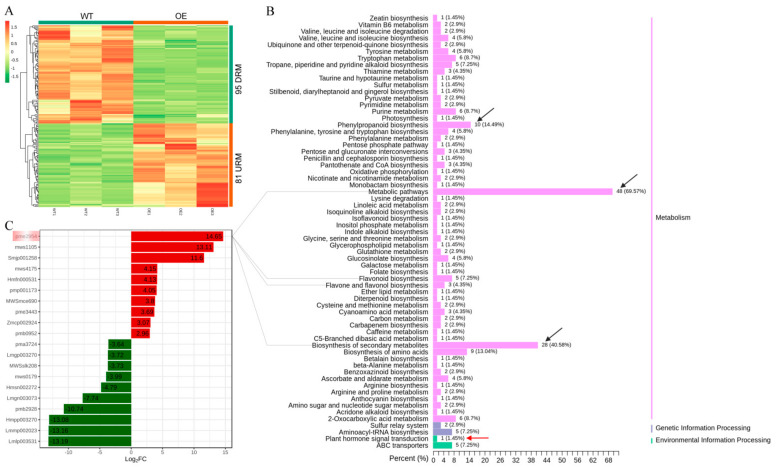
Multiple analysis of SDM (Significantly Different Metabolites). (**A**) Hierarchical clustering of SDMs. The *x*-axis and *y*-axis represent the specific sample name and SDM, respectively. Different colors in the heatmap represent the relative content of SDM. Red and green colors represent high and low content, respectively. The WT and OE in the note bar above the heatmap represent wild type and overexpression, respectively. The URM and DRM in the note bar on the right side of the heatmap denote up-regulated metabolites and down-regulated metabolites, respectively. (**B**) KEGG (Kyoto Encyclopedia of Genes and Genomes) classification of SDM. *x*-axis represents the number and percentage of enriched metabolites in KEGG pathways, while the *y*-axis represents specific names of KEGG pathways. The black arrows indicate the top 3 KEGG pathways with more number of SDMs. The red arrow indicates the plant hormone signal transduction pathway. (**C**) The top 10 SDM in WT vs. OE comparison group. The *x*-axis and *y*-axis represent Log_2_FoldChange and specific SDM index, respectively. Pme2954, quercetin; mws1105, gramine; Smjp001258, abrine; mws4175, D-glucurono-6,3-lactone; Hmfn000531, L-ascorbic acid (vitamin C); pmp001173, lyciumin A; MWSmce690, erythorbic acid, isoascorbic acid; pme3443, sinapinaldehyde; Zmcp002924, cyanidin-3-O-(2″-O-glucosyl)glucoside; pmb0952, thiamine (vitamin B1); pma3724, 1-O-feruloylquinic acid; Lmgp003270, scopoletin-7-O-glucuronide; MWSslk208, kaurenoic acid; mws0179, chlorognic acid methyl ester; Hmsn002272, demethyl coniferin; Lmgn003073, 5-O-feruloylquinic acid; pmb2928, gallic acid-4-O-glucoside; Hmpp003270, luteolin-4′-O-glucoside; Lmmp002023, caffeoylspermine; Lmlp003531, luteolin-3′-O-glucoside; and Pme2954 with the largest difference was noted with pink gradient box. The grey lines indicate specific pathways for Pme2954 metabolite.

We carried out the correlation and PCA between different groups of samples to ensure the quality of samples and the accuracy of the whole analysis process (Figures S1 and S2). A total of 1000 metabolites detected by the UPLC-MS/MS platform combined with self-building databases were classified into 13 categories including flavonoids, lipids, phenolic acids, alkaloids, others, amino acids and derivatives, organic acids, nucleotides and derivatives, lignans and coumarins, terpenoids, quinones, tannins, and steroids. The abundance of flavonoids in our analysis indicated the role of AaMYBC1 TF involved in the flavonoid metabolism pathway (Tables S1 and S2). In addition, the hierarchical cluster analysis of metabolites showed that WT and OE were clustered together (Figure S3). Additionally, multiple clustering analysis of SDM (Significantly Different Metabolites) was conducted to clarify the effects of overexpression of *AaMYBC1* on differentially accumulated metabolites. The analysis resulted in the identification of 81 URM (Up-Regulated metabolites) and 95 DRM (Down-Regulated metabolites) (Figure 2A and Table S3), indicating that overexpression of AaMYBC1 had an important impact on the growth and development of related metabolites in tobacco internodes. Based on KEGG annotations, the SDM were classified as metabolism, genetic information processing, and environment information processing related metabolites, and metabolism was the largest class that contained most of SDM. Additionally, metabolic pathways, biosynthesis of secondary metabolites and phenylpropanoid biosynthesis were the top 3 pathways found in the metabolism class (Figure 2B). The pme2954 (quercetin) was found to be the most significant URM involved in metabolic pathways, flavonoid biosynthesis, flavone and flavonol biosynthesis, and biosynthesis of secondary metabolites (Figure 2C). Our results confirmed the key role of AaMYBC1 in flavonoid metabolism, which was consistent with our previous study that AaMYBC1 was a flavonoid-related TF in *Actinidia arguta* [57,58]. It is well known that phytohormones are key regulators of cell elongation or size. Interestingly, pme1651 (Indole 3-acetic acid, IAA) was the only SDM found in the plant hormone signal transduction pathway, which suggested IAA might be the key phytohormone regulating internode elongation.

### 3.3. RNA-Seq Data Overview and DEG Identification

**Figure 3:**
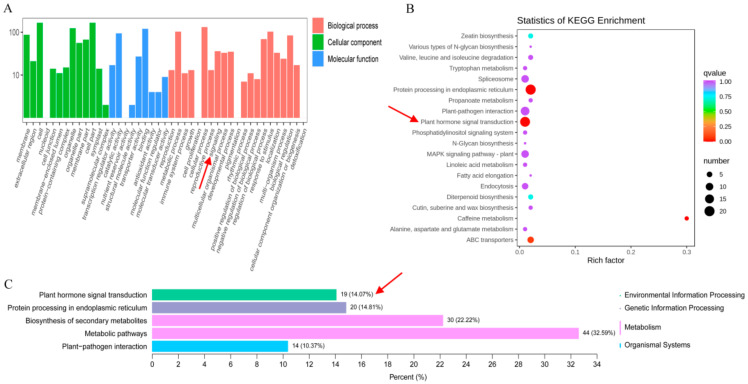
GO (Gene Ontology) and KEGG classification of DEGs. (**A**) GO classification (biological process, cellular component, and molecular function) of DEGs. The *x*-axis represents the GO terms, and the *y*-axis represents the number of DEGs involved in corresponding terms. (**B**) Statistics of KEGG pathway enrichment. The *x*-axis represents the rich factor and the *y*-axis represents the specific KEGG pathways. The size of circle denotes number of DEGs in a KEGG pathway, and the color bar indicates the significance level of each enriched pathway. (**C**) Statistics of top five KEGG pathways. The *x*-axis represents number of DEGs involved in the pathway and the percentage of DEGs annotated to this pathway to the total number of annotated DEGs. The *y*-axis represents the specific KEGG pathways. The red arrow indicates the plant hormone signal transduction pathway.

Sequencing quality control is the premise of obtaining reliable transcriptome data. Therefore, we used the distribution of sequencing error rate and GC contents (Figures S4 and S5), to obtain clean reads by removing low-quality reads and adapters (Figure S6). The clean reads accounted for >96% of each sample, which ensured the quality and reliability of the data. Finally, we obtained a total of 40.83 Gb clean data, and the percentage of Q30 and Q20 bases was 94% and 98%, respectively (Table S4). Additionally, our results showed that 96% of total reads were successfully mapped to the reference genome (Table S5). We identified a total of 446 DEGs (differential expression genes) were identified in OE vs. WT comparison, in which 286 and 160 were up-regulated DEGs and down-regulated DEGs, respectively (Figure S7 and Table S6). GO term analysis resulted in classification of GO terms into three groups: biological process, cellular component and molecular function. A total of 176 DEGs were assigned to the biological process, 199 DEGs were assigned to the cellular component, and 189 DEGs were assigned to molecular function (Figure 3A and Table S7). Similarly, we performed the KEGG enrichment analysis for DEGs and the results showed that a total of 135 DEGs were assigned to 59 KEGG pathways (Figure 3B and Table S8), of which the top five pathways included metabolic pathways, biosynthesis of secondary metabolites, protein processing in the endoplasmic reticulum, plant hormone signal transduction and plant-pathogen interaction (Figure 3C).

### 3.4. Screening of Candidate Gene

**Figure 4:**
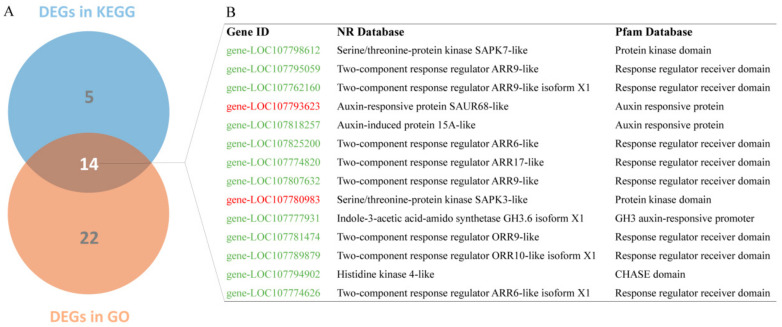
Screening of DEGs involved in plant hormone signal transduction. (**A**) The Venn diagram analysis of plant hormone signal transduction related DEGs from KEGG and GO analysis. (**B**) The annotation information of 14 common DEGs from NR and Pfam online databases. Red and green color genes represent up-regulated and down-regulated DEGs, respectively.

Earlier in this study, we mentioned that the phytohormones are key regulators of cell elongation. Therefore, we emphasized the plant hormone signal transduction pathway to screen the candidate genes by combining KEGG pathway enrichment and GO enrichments results. A total of 14 DEGs were identified as candidate genes that were possibly involved in the hormone signal transduction (Figure 4A), of which 2 and 14 were up-regulated and down-regulated DEGs, respectively. These DEGs were mainly annotated as protein kinase, response regulator receiver, auxin-responsive protein, GH3 auxin-responsive promoter, CHASE, etc., while most of them were annotated as auxin-related genes, implying that auxin signaling might play a key role in internode elongation (Figure 4B).

### 3.5. Combined Analysis of Metabolome and Transcriptome

**Figure 5:**
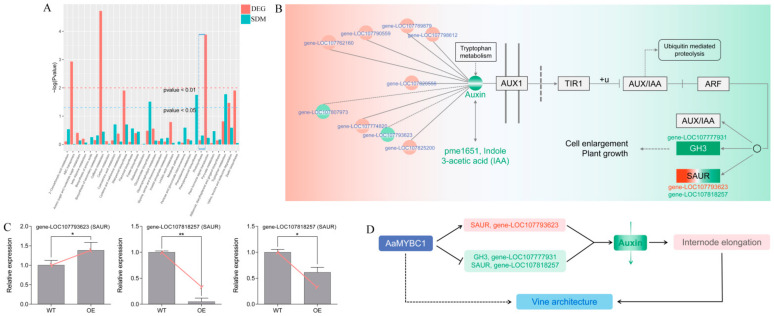
Regulatory mechanism of internode elongation based on combined analysis of metabolome and transcriptome. (**A**) Integrative KEGG pathway of DEGs and SDMs. The *x*-axis represents the metabolic pathway. The *y*-axis represents −log (*p*-value) of DEGs indicated by red color bars and SDMs indicated by green color bars. ‘Plant hormone signal transduction’ is indicated by a blue dotted box. (**B**) Auxin signal transduction pathway including DEGs and SDMs. pme1651, annotated as IAA, was the unique auxin-related SDM detected in OE vs. WT comparison group. A total of nine DEGs interacted with pme1651. After a series of analyses, three DEGs including gene-LOC107793623, gene-LOC107777931 and gene-LOC107818257 were identified, which played a key role in the regulation of internode length. (**C**) The RT-qPCR validation of three genes including gene-LOC107777931, gene-LOC107793623 and gene-LOC107818257. Values are presented as mean ± SD for three biological replicates. Statistical significance was presented as ** *p* < 0.01, * *p* < 0.05. The solid red line represents the expression trend of three genes from transcriptome data. (**D**) A simple model of AaMYBC1 induced internode elongation by mediating the expression of auxin-related genes, and regulation of vine architecture.

A combined analysis of metabolome and transcriptome was performed to explore the related DEGs and SDMs involved in the same KEGG pathway. A total of 30 integrative KEGG pathways were found to have DEGs and SDMs associated with each other. The top three KEGG pathways with the most number of genes were metabolic pathways with 44 DEGs, biosynthesis of secondary metabolites with 30 DEGs, and plant hormone signal transduction pathway with 19 DEGs (Figure 5A and Table S9), indicating that overexpression of *AaMYBC1* could control some biological phenotypes mainly by affecting plant metabolism and hormone signal transduction. The involvement of phytohormones, particularly IAA, compelled us to focus on the hormone signal transduction pathway. Our results showed that a total of nine DEGs (seven URDEGs and two DRDEGs) interacted with pme1651 (Indole 3-acetic acid, IAA), which was the only phytohormone detected in metabolome analysis. Results from the further analyses showed that the phenotype was mainly associated with changes in the expression of three genes among which gene-LOC107777931 belonged to GH3 family, gene-LOC107793623, and gene-LOC107818257 belonged to SAUR family (Figure 5B). To confirm the reliability of transcript level of these three genes from transcriptome data, we carried out the RT-qPCR analysis that showed the gene expression level was consistent with the transcriptome results (Figure 5C and Tables S10 and S11). At last, we deduced the model of AaMYBC1-mediated internode elongation, that is, overexpression of AaMYBC1 can lead to the expression changes of three genes, including the increase of SAUR gene-LOC107793623 and the decrease of GH3 gene-LOC107777931 and SAUR gene-LOC107818257, to reduce the content of auxin and finally lead to internode elongation (Figure 5D). At present, the relevant measures to improve tree architecture mainly depend on cultivation managements including tree shaping and pruning [59], integrated pattern of water and fertilizer [60], flowers and fruits thinning, etc. There are relatively few studies available for tree architecture improvement at the molecular level. Therefore, it is worthwhile for breeders to discover the key genes regulating plant architecture. The AaMYBC1 regulates internode length by mediating the expression of auxin-related genes, which suggests the potential function of AaMYBC1 in improving kiwifruit architecture. While the specific interaction mechanism between AaMYBC1 and AaSAUR or AaGH3, and the process of auxin signal transduction mediated by AaMYBC1 both need to be further explored, from preliminary evidence, we speculate that AaMYBC1 forms a regulatory module by binding to the upstream promoter region of *AaSAU*R and *AaGH3* genes to activate or repress the expression of targets. This regulatory module can mediate the change in auxin level and affect internode length, which plays a potential role in shaping vine architecture. Therefore, the cis-element analysis to predict MYB binding sites in the promoter region of *AaSAU*R and *AaGH3* genes will be useful to gain insights into the regulatory module. In addition, exploring molecular regulatory networks underlying internode length and vine architecture must rely on the genomic information of genes, however, no specific genome of *Actinidia arguta* has been published so far due to the presence of tetraploid in the genome. Other omics studies about fruit quality on *Actinidia arguta* have been reported [57], which would provide an invaluable source of information to better understand the complex trail of fruit quality and plant architecture.

## 4. Conclusions

As a functional regulator, AaMYBC1 regulated the internode elongation of transgenic tobacco plants mainly by mediating the auxin signal transduction pathway. Three auxin-related genes including two SAUR genes and one GH3 gene were screened as candidate genes to perform a function study. Our results proved the potential role of AaMYBC1 in improving vine architecture by regulating internode length. Current findings provided a new perspective for relevant research on the shaping the architecture of perennial fruit trees.

## Data Availability

Not applicable.

## Supplementary Materials

The following supporting information can be downloaded at: https://www.mdpi.com/article/10.3390/genes13050817/s1, Figure S1: The correlation analysis between different samples; Figure S2: The PCA (Principal Component Analysis) of samples; Figure S3: The cluster heatmap analysis of all samples and metabolites; Figure S4: Distribution of sequencing error rate of six samples including OE1, OE2, OE3, WT1, WT2 and WT3; Figure S5: Distribution of GC content of six samples including OE1, OE2, OE3, WT1, WT2 and WT3; Figure S6: Statistics of raw sequencing data of six samples including OE1, OE2, OE3, WT1, WT2 and WT3; Figure S7: The cluster heatmap analysis of samples and DEGs (differentially expressed genes); Table S1: Statistics of metabolites; Table S2: The specific information of 1000 detected metabolites; Table S3: The specific information of 176 SDMs; Table S4: Statistics of transcriptome assembly; Table S5: Statistics of reads mapped reference genome; Table S6: The specific annotations and expression information of 446 DEGs; Table S7: The GO enrichment result of DEGs; Table S8: KEGG enrichment result of DEGs; Table S9: Association pathway results of DEGs and SDMs; Table S10: The primers used for RT-qPCR; Table S11: The original data of RT-qPCR for four genes.
