# Supplementary material for: Novel Role of AaMYBC1 in Regulating Actinidia arguta Vine Architecture by Elongating Internode Based on Multi-Omics Analysis of Transgenic Tobacco
Source: Genes (Basel). 2022 May 3;13(5):817. doi: 10.3390/genes13050817 (PMC9140693; doi:10.3390/genes13050817)
Supplement: Supplementary file 1 [file genes-13-00817-s001.zip › Supplementary Figures.pdf]

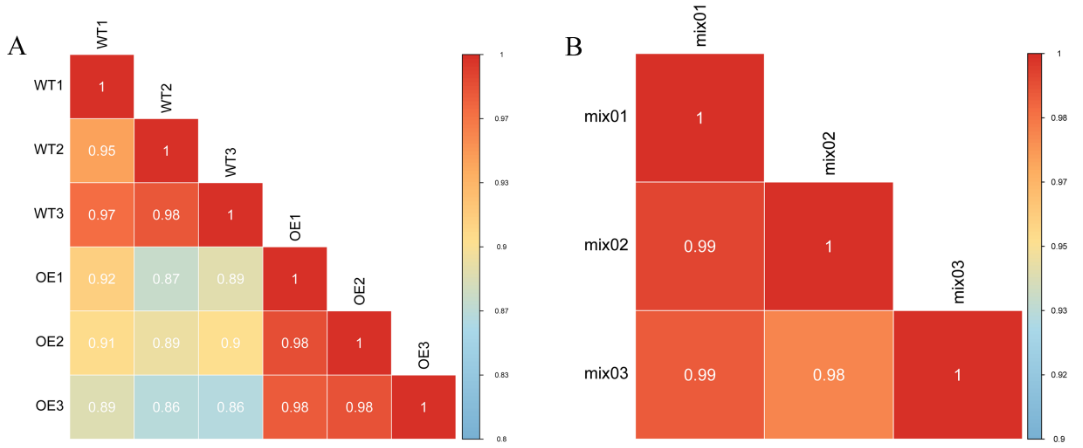

Figure S1. The correlation analysis between different samples. (A) Repeated correlation assessment of experimental samples; (B) Repeated correlation assessment of QC (Quality Control) samples. The different samples including experimental samples (WT1, WT2, WT3, OE1, OE2, OE3) and QC samples (mix01, mix02, mix03) were noted at longitudinal and diagonal lines. Different color squares represent different Pearson correlation coefficients. The more red the color, the stronger the correlation, and the more blue the color, the weaker the correlation. The specific correlation coefficient is marked in the squares.

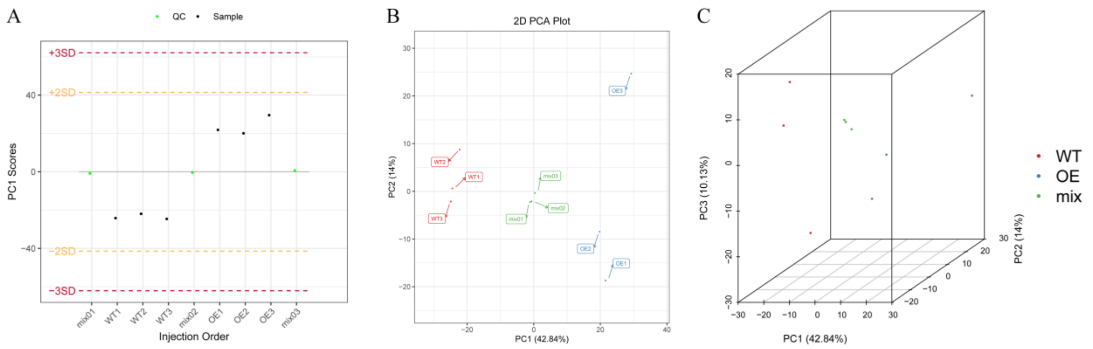

Figure S2. The PCA (Principal Component Analysis) of samples. (A) PC1 control of overall samples that is a statistical process control of PC univariate. The horizontal and vertical coordinates represent sample detection sequence and the PC1 value, respectively. The yellow and red lines dotted lines define the range of positive and negative two or three standard deviations, respectively. Black and green dots represent experimental and QC samples, respectively. (B) The two dimensional PCA of overall samples. (C) The three dimensional PCA of overall samples. PC1, PC2 and PC3 represent the first, second and third principal components, respectively. The percentage represents the interpretation rate of the principal component to the data set. Each point in the figure represents a sample, and the samples from the same group are indicated by the same color.

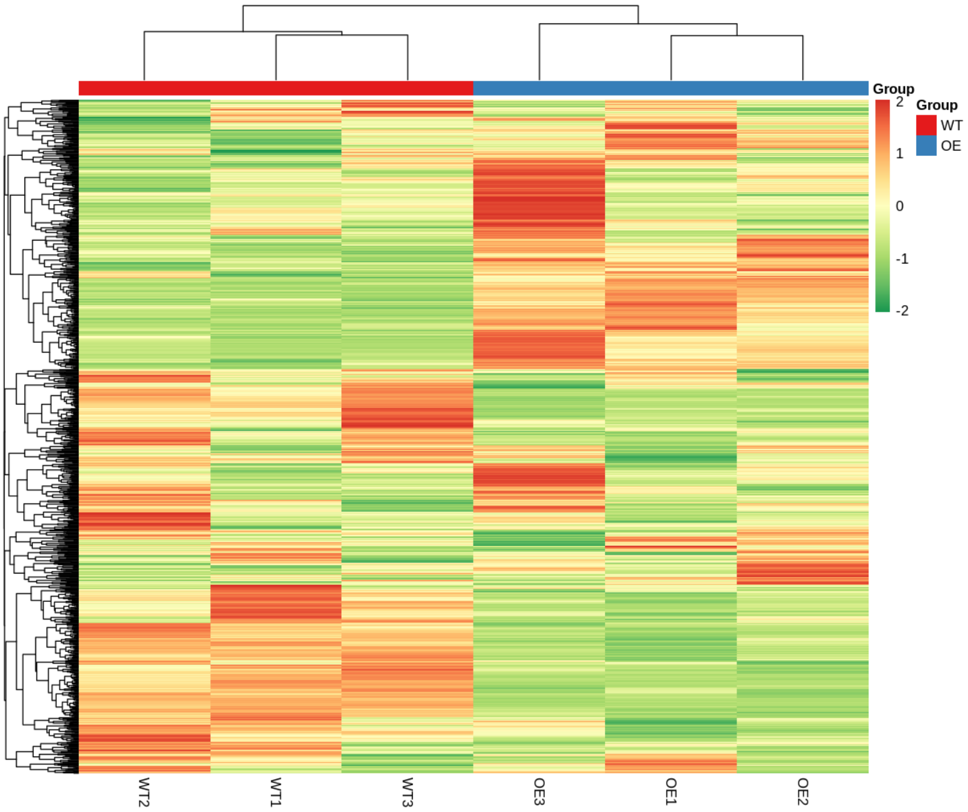

Figure S3. The cluster heat map analysis of all samples and metabolites. The horizontal and vertical coordinates represent the specific sample name and metabolite, respectively. Different colors are the values obtained after relative content standardization. Red and green colors represent high and low content of metabolites, respectively.

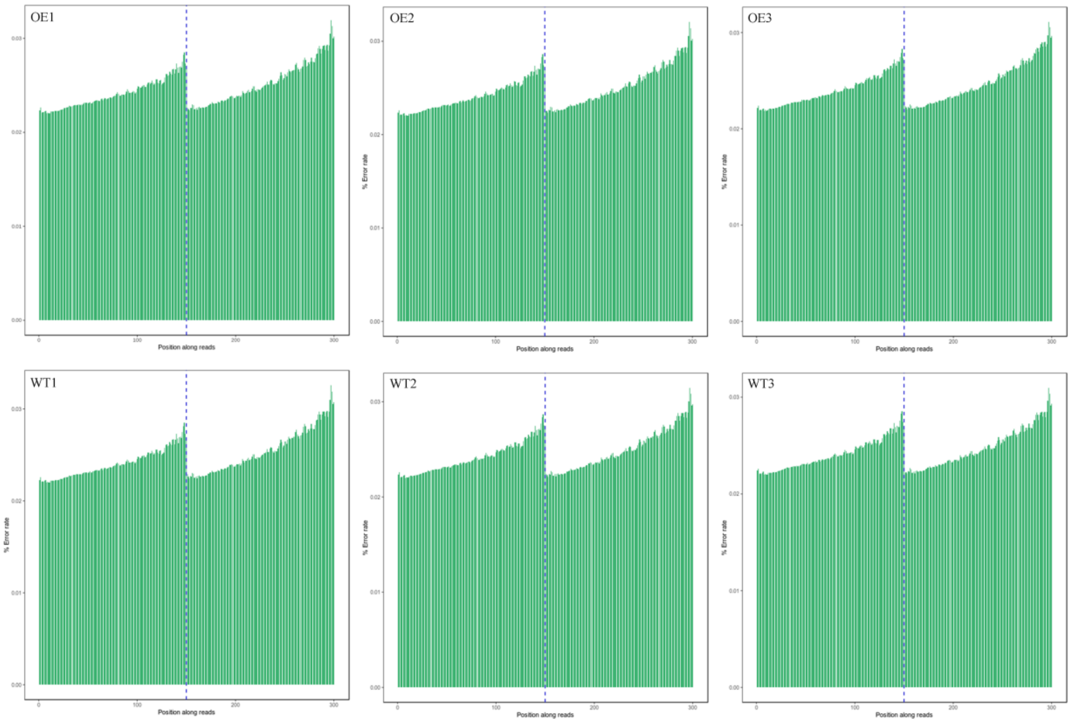

Figure S4. Distribution of sequencing error rate of six samples including OE1, OE2, OE3, WT1, WT2 and WT3. The horizontal and vertical coordinates represent base position of reads and error rate of single base.

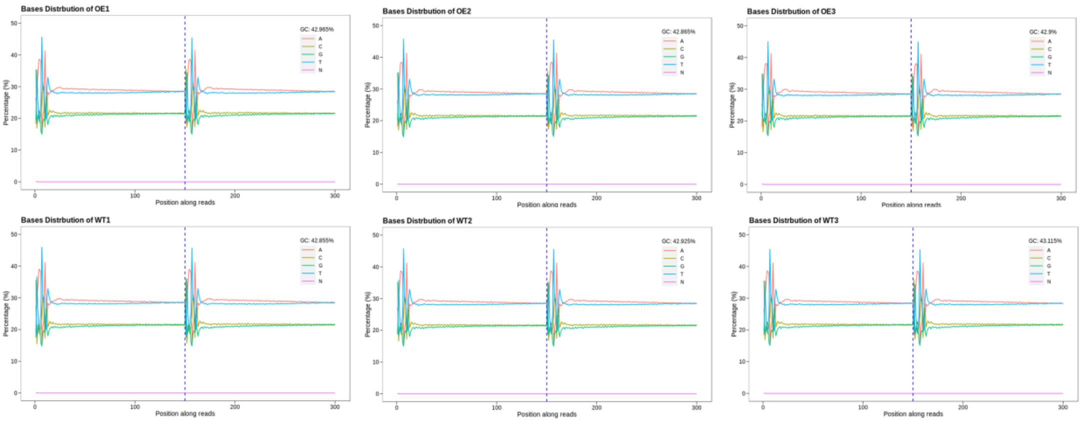

Figure S5. Distribution of GC content of six samples including OE1, OE2, OE3, WT1, WT2 and WT3. The horizontal and vertical coordinates represent base position of reads and percentage of single base.

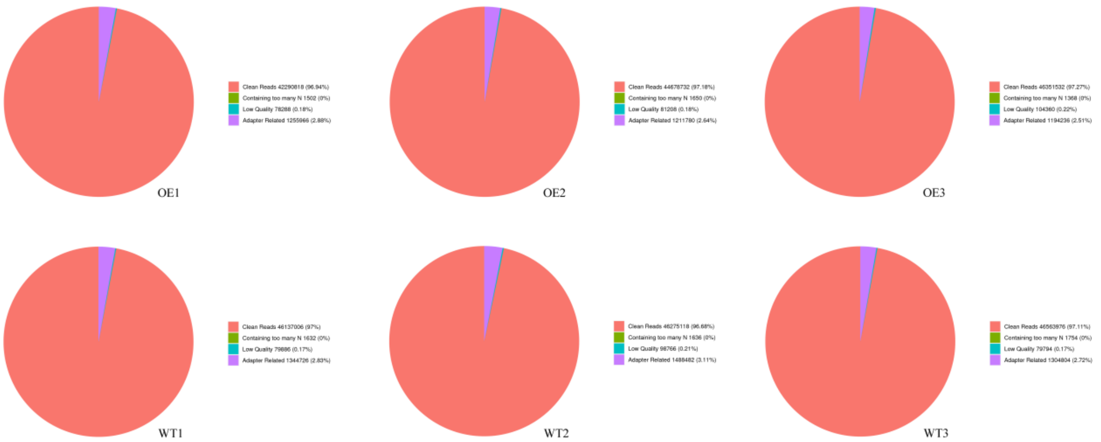

Figure S6. Statistics of raw sequencing data of six samples including OE1, OE2, OE3, WT1, WT2 and WT3. The statistical components include clean reads, containing N, low quality and adapter related.

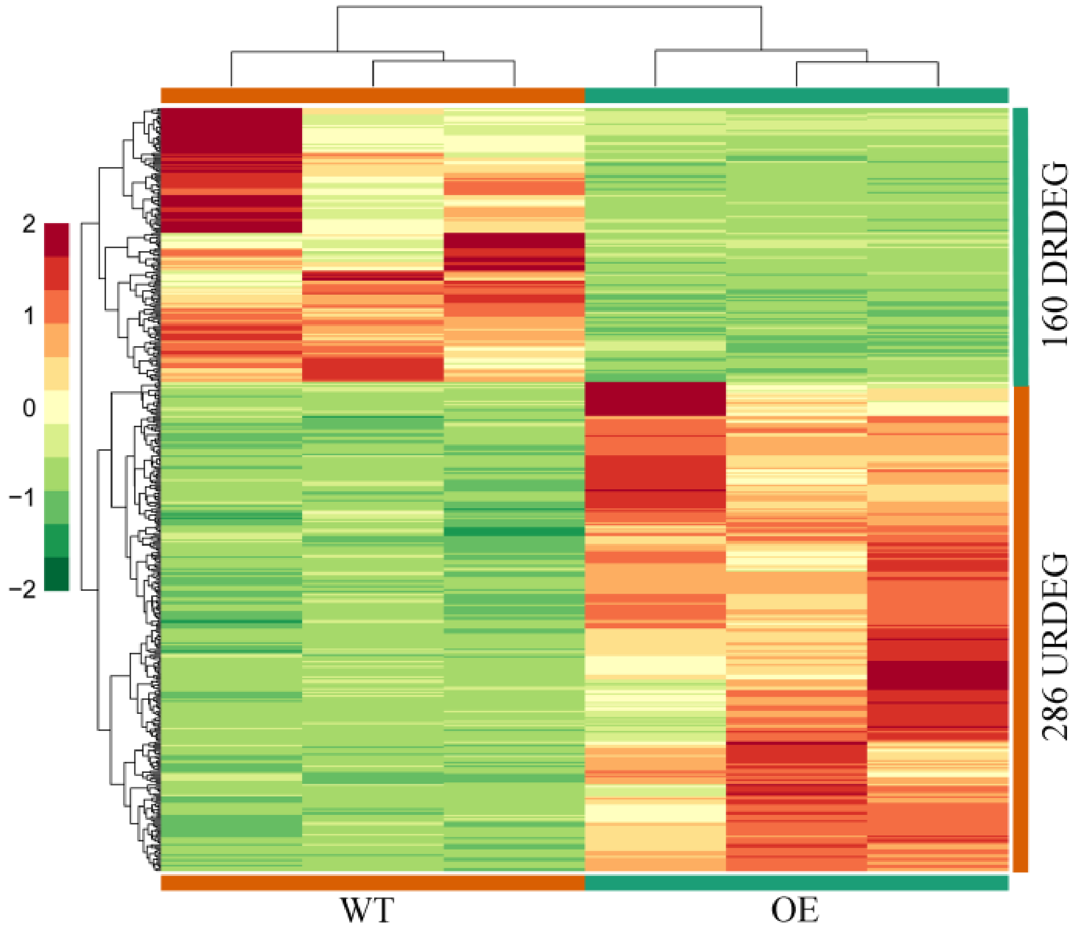

Figure S7. The cluster heat map analysis of samples and DEGs (differentially expressed genes). URDEG, up-regulated DEGs; DRDEG, down-regulated DEGs. The horizontal and vertical coordinates represent sample name and DEGs, respectively. Different colors are the values obtained after relative expression standardization. Red and green colors represent high and low expression level, respectively.
