# Supplementary material for: Novel Role of AaMYBC1 in Regulating Actinidia arguta Vine Architecture by Elongating Internode Based on Multi-Omics Analysis of Transgenic Tobacco
Source: Genes (Basel). 2022 May 3;13(5):817. doi: 10.3390/genes13050817 (PMC9140693; doi:10.3390/genes13050817)
Supplement: Supplementary file 1 [file genes-13-00817-s001.zip › Table S1.pdf]

Table S1 Statistics of metabolites.

Notes: WT, wild typed samples. OE, over expressed samples. SDM, significantly differential metabolites. URM, up regulated metabolites. DRM, down regulated metabolites.

Group name		All metabolites	All SDM	URM	DRM
WT vs OE		1000	176	81	95
Class I categories	Flavonoids	208	30	13	17
	Lipids	146	8	1	7
	Phenolic acids	138	37	14	23
	Alkaloids	118	40	16	24
	Others	104	17	12	5
	Amino acids and derivatives	76	12	8	4
	Organic acids	72	6	1	5
	Nucleotides and derivatives	51	10	10	0
	Lignans and coumarins	44	11	4	7
	Terpenoids	29	4	2	2
	Quinones	9	0	0	0
	Tannins	4	1	0	1
	Steroids	1	0	0	0
