# Supplementary material for: Novel Role of AaMYBC1 in Regulating Actinidia arguta Vine Architecture by Elongating Internode Based on Multi-Omics Analysis of Transgenic Tobacco
Source: Genes (Basel). 2022 May 3;13(5):817. doi: 10.3390/genes13050817 (PMC9140693; doi:10.3390/genes13050817)
Supplement: Supplementary file 1 [file genes-13-00817-s001.zip › Table S4.pdf]

Table S4 Statistics of transcriptome assembly.

Notes: WT, wild typed samples; OE, over expressed samples; Q20, percentage of base with Qphred value not less than 20 in total base; Q30, percentage of base with Qphred value not less than 30 in total base;

Sample	Raw reads	Clean reads	Clean base (G)	Error rate (%)	Q20 (%)	Q30 (%)	GC content (%)
OE1	43,626,574	42,290,818	6.34	0.02	98.27	94.45	42.97
OE2	45,973,370	44,678,732	6.7	0.02	98.26	94.42	42.86
OE3	47,651,496	46,351,532	6.95	0.02	98.38	94.75	42.9
WT1	47,563,250	46,137,006	6.92	0.02	98.24	94.35	42.86
WT2	47,864,002	46,275,118	6.94	0.02	98.32	94.57	42.92
WT3	47,950,328	46,563,976	6.98	0.02	98.38	94.68	43.11
